# Supplementary material for: Are carbon emissions trading and green financial instruments synergistic? -Comprehensive quantitative research based on content analysis
Source: PLoS One. 2024 Mar 7;19(3):e0298601. doi: 10.1371/journal.pone.0298601 (PMC10919601; doi:10.1371/journal.pone.0298601)
Supplement: S1 Data — (ZIP) [file pone.0298601.s001.zip › Data/地级市Z_F工作报告.zip!/╕╩╦α/╛╞╚¬╩╨/└┤╫╘╣1⁄2╓┌║┼í╛┬φ┐╦╩2╛▌═°í┐_2011.docx]

各位代表：

　　现在，我代表酒泉市人民政府向大会作工作报告，请予审议，并请各位政协委员和列席人员提出意见。

　　2010年工作回顾

　　2010年，全市各级政府在中共酒泉市委的正确领导下，以科学发展观为统揽，认真贯彻党的十七届四中、五中全会精神，深入实施省委、省政府区域发展战略和市委"两抓整推"工作思路，攻坚克难，扎实工作，较好地完成了市二届人大六次会议确定的各项任务。预计全市生产总值达到405亿元，增长18%；全社会固定资产投资438亿元，增长46%；财政收入达到50.5亿元，增长12.2%，财政支出65.7亿元，增长32.3%；社会消费品零售总额91亿元，增长20%；城镇居民人均可支配收入15076元，增长10%，当地农民人均纯收入7180元，增长12%，移民人均纯收入1838元，增长20%；人口自然增长率控制在2.85‰；节能减排完成约束性指标；居民消费价格指数上涨4.5%；政府承诺的创业促就业、安全饮水等8件惠民实事有6件全面完成，廉租住房建设和农村危旧房改造已完成工程量的85%，部分跨年工程可在今年上半年竣工。

　　1、抢抓政策机遇，项目建设加快跟进。紧紧抓住新一轮西部大开发战略和国扶甘肃47条意见的政策机遇，对关乎酒泉发展的20个重大课题进行调研，加紧申报成熟项目，加快建设重点项目，谋划储备后续项目，项目建设呈现出投资大、进度快、资金到位好的特点。全市开工建设项目1107项，其中：投资亿元以上项目72项，5000万元以上项目160项，千万元以上项目472项，完成市县属固定资产投资398亿元，增长60%。基础设施建设取得新突破，瓜星高速、敦当公路、兰新铁路第二双线酒泉段和敦煌公路客运枢纽站开工建设，敦格铁路完成初设审查，酒航铁路获国务院和中央军委批复，酒嘉城际一级公路、肃北至阿克塞二级公路、酒泉公路客运枢纽站完成前期工作。招商引资规模扩大，组团参加西洽会、兰洽会和西博会，举办国际新能源产业博览会和第八届银企合作项目推介会，全市签约招商引资合同项目136个，开工102个，签约金额357亿元，其中到位资金160亿元，增长86%。

　　2、突出风电开发，"6+2"产业迅速崛起。以风电开发为牵引，全面实施振兴工业"6＋2"行动计划，工业增速明显加快，预计完成工业增加值173亿元，增长35%。千万千瓦级风电基地建设取得重大进展，一期516万千瓦装机任务超额完成，新增风电装机330万千瓦，累计达到550万千瓦，二期800万千瓦风电项目全面启动，我市被评为全国最具投资价值的新能源产业城市，玉门、瓜州分别名列"新能源产业百强县"第二、第五位。建立风电预报中心，完成风能资源详查报告。编制光电产业发展规划，敦煌、金塔、玉门光电工业园开工建设，在建和建成光电装机170兆瓦。调峰电源建设加快推进，酒泉热电厂2×33万千瓦项目进展顺利，明沙窝、常乐、红柳洼电厂完成可研报告。750千伏电网联网工程建成投运，实现了西北电网与新疆电网互联互通，编制风电基地二期输电规划，±800千伏特高压直流输变电工程完成选址。按照"资源换设备、总装带配套、装备促发展"的思路，加快风光电装备制造业发展，形成年产风机2000套、3000兆瓦生产能力，实现销售收入223亿元，是2009年的3.5倍。编制完成高载能产业发展规划和矿产资源勘查开发规划，推进金、钒等矿产资源整合。加快水能资源开发，柳沟峡80万千瓦抽水蓄能电站完成选址，新增水电装机4万千瓦，累计装机达到26万千瓦，在建水电装机30万千瓦。核乏燃料处理项目完成厂址勘测和可研报告，4×60万千瓦核电项目列入国家规划。

　　3、坚持"一特四化"，现代农业扎实推进。继续加大"三农"扶持力度，财政"三农"支出13.25亿元，增长15%，发放粮食、良种、农资等惠农补贴2.48亿元，农民人均受益477元。大力发展节水高效特色农业，调整优化种植结构，新增蔬菜、瓜果、药材等2000元以上高效田21万亩，累计达到162万亩。加强粮食储备体系建设，新增粮食储备8500吨。狠抓牛羊产业大县建设，提高草食畜牧业比重，新建养殖小区220个，累计达到889个，新增万元规模养殖户1920户，累计达到1.99万户，秸秆加工转化利用率达到60%，牛、羊出栏率分别增长9.3%和10.2%。扶持发展龙头企业，新建、扩建农业产业化项目21个，规模以上龙头企业达到85家，辐射带动生产基地165万亩，占到播种面积的74%，农产品贮藏加工增值率达到49.7%，提高4.7个百分点。新增农民专业合作社136个，累计达到312个，农业组织化程度进一步提高。推进标准化生产，认定无公害农产品产地54个，认证无公害农产品62个，"玉门酒花"被评为国家地理标志保护产品。拓宽培训渠道，采取订单培训、校企联合培训和项目定向培训等形式，培训农村劳动力5万人，输转农村劳动力14.9万人次，实现劳务收入13.6亿元。推进农村土地流转，新增流转面积4.9万亩，累计达到23万亩，占家庭承包耕地面积的13%。扎实推进新农村建设，科学编制乡村建设规划，建立村容村貌整治示范点160个，新建社区化中心村20个。

　　4、改善市政设施，城市功能明显提升。充分发挥规划在城市建设中的导向作用，制定城乡规划一体化实施意见，市域城镇体系规划完成文本编制，城市总体规划加紧编制，历史文化名城保护规划通过初审。完成城市建设投资22.2亿元，增长52%，是"十一五"时期投资最多的一年。完善酒泉市区道路、供水、供热、排污等市政设施，完成金泉路、盘旋中路等8条道路建设工程，建成西大街地下人行通道、城区防洪排涝工程、医疗废物处置中心，完成城市供水分户改造3200户、供热计量改造47.3万平方米，酒泉北大河生态治理项目上报国家发改委待批。积极创建园林城市，新增绿地111万平方米，城区绿化覆盖率达到35%，酒泉市区及金塔、敦煌、玉门通过省级园林城市验收，阿克塞荣获国家园林县城称号。创新城市管理机制，规范市政设施、园林绿化、环卫保洁标准，组建"数字城管"监控中心。建立城市供热应急预案，修改完善城市供热管理及监督办法。深入开展全国文明城市创建活动，狠抓城中村、城市出入口和城乡结合部环境整治，城市及周边环境得到净化。

　　5、扩大消费需求，商贸旅游繁荣活跃。认真落实国家扩大消费、稳定出口、支持服务业发展的政策措施，第三产业完成增加值140亿元，增长12%，对经济增长的贡献率达到30.5%，我市被确定为全省唯一的国家服务业综合改革试点城市。加快市场体系建设，时代购物中心、昌兴市场及瓜州元通市场建成运营。扩大农村消费市场，改造农家店215个，发展农资配送中心27个，兑付"家电下乡"和汽车、摩托车购置补贴4749万元。扶持创汇企业和出口产品基地建设，全市预计完成进出口总额6000万美元，其中出口4440万美元。进一步壮大旅游产业，实施旅游项目22个，莫高窟游客接待中心、瓜州锁阳城景区项目开工建设，完成雅丹地质公园基础设施建设项目。提升旅游服务质量，新增星级饭店3家，酒泉宾馆荣获"中国饭店金星奖"，9家饭店荣获"全省优秀旅游星级饭店"称号。积极拓展旅游市场，举办敦煌飞天文化节和金塔金秋胡杨节，开展"旅游大篷车千里行"活动，协调开通上海至敦煌航班，增开敦煌至兰州、乌鲁木齐至敦煌列车。接待海内外游客405万人次，增长16%，实现旅游综合收入37.7亿元，增长60%，占全市GDP的比重达到9.3%，我市荣获"中国最具国际影响力旅游目的地"称号。

　　6、强化环境治理，生态建设取得实效。把节水作为生态建设的关键，编制完成百万亩农业节水规划，党河、洪临灌区节水改造工程建成，全市新增节水面积39.9万亩，其中膜下滴灌3.4万亩，节水灌溉面积达到148万亩。严格落实取水总量控制与用水定额管理制度，农业水权证全部发放到户，新装智能水表3153块，全面完成万眼机井智能化管理任务。继续实施封滩育林、退耕还林、退牧还草等生态建设工程，敦煌被列为全省首个国家级可持续发展试验区。广泛开展植树造林活动，完成人工造林11.8万亩，全民义务植树410万株，集体林权制度改革顺利推进。认真落实国家淘汰落后产能政策和甘肃省循环经济总体规划，开展能效达标、节能评估和"双超双有"企业强制性清洁生产审核，对重点用能企业进行严格监控，依法关停小冶炼企业12户，淘汰铁合金行业落后产能1.8万吨，全市单位GDP能耗下降4%。完善污染减排监测体系，重点污染源全部安装自动监控装置，酒泉污水处理厂改造升级工程建成运行。敦煌、玉门污水处理厂完成整改，祁药集团土霉素厂、玉门炼化污水处理厂完成技术改造，新增二氧化硫削减能力150吨、化学需氧量削减能力2000吨。编制完成资源型城市可持续发展规划，建成水选改性纤维、建筑废料利用等新型建材企业84户，玉门建化工业园循环经济发展规划通过专家评审。高度重视再生资源体系建设，我市区域性大型再生资源回收利用基地项目获国家批准。积极创建国家环保模范城市，公众对城市环境保护满意率达到86.4%，比2009年提高2.2个百分点。

　　7、加大资金投入，社会事业协调发展。启动科技创新八项行动，组织实施科技专项120项。提高科技成果产出水平，5项成果获得省科技进步一、二、三等奖，申请国家专利196件，授权114件。新增甘肃著名商标12件，14项产品荣获甘肃名牌称号，科技进步对经济增长的贡献率达到50%。组建酒泉风光电装备制造生产力促进中心，酒泉工业园区被认定为国家风电装备高新技术产业化基地和省级高新技术产业开发区。加快教育资源整合，调整撤并农村中小学102所，农村中小学布局结构调整任务基本完成。开工建设中小学校舍安全工程37个，酒泉四中、五中和46座寄宿制学校公共浴室建成使用。组建全国首所新能源技术学院，酒泉职业技术学院示范性高职院校重点项目通过省级验收。全面落实义务教育经费保障和"两免一补"政策，落实公用经费和补助资金1.37亿元，教科书免费发放覆盖面达到85%。医药卫生体制改革稳步推进，制定公共卫生服务均等化实施、考核办法，为城乡居民免费提供健康教育、传染病防治等9项基本公共卫生服务。全市乡镇、村、社区卫生服务机构实现基本药物"零差价"，基层医疗机构药品费用下降45.6%。狠抓医疗卫生项目建设，市医院门诊大楼、市二院标准化建设项目完成主体工程，4家县市医院标准化建设项目开工。为71个乡镇卫生院配备救护车，86%的村卫生室实行乡村一体化管理。提高公共卫生应急保障能力，建成市级突发公共卫生事件应急物资库，成功处置"6.12"鼠疫疫情，农村卫生紧急救援服务网络覆盖率达到95%。深化人口与计划生育综合改革，统筹解决人口问题，我市被评为全国人口计生综合改革和婚育新风进万家活动示范市。开工建设文化设施项目36个，建成文化信息资源共享工程乡村服务点467个，新建"农家书屋"28个，累计达到463个，"六馆合一"的酒泉文化中心完成设计方案招标。第三次文物普查通过省上验收，新发现文物点666处，敦煌、玉门、阿克塞博物馆主体工程完工。《酒泉通史》完成初稿，肃州、瓜州、金塔地方志正式出版。认真筹划和精心组织全省第七届民运会和第八届残运会，广泛开展全民健身活动。广播电视"村村通"工程完成年度任务，肃北、阿克塞两县列入国家广播电视"西新工程"，玉门、瓜州数字电视转换全面启动。高度重视救助、助残工作，市儿童福利院和玉门、瓜州、敦煌社会福利中心建成使用，市流浪儿童救助保护中心开工建设，残疾人综合服务中心、盲人有声读物阅览室、阳光家园等项目相继建成。深入开展安全生产检查，关闭不具备安全生产条件的网点646个。按照"发现得早、化解得了、控制得住、处理得好"的要求，实行信访事项会商会诊、信访初始责任追究、重大决策信访评估制度，化解信访积案115件，调处矛盾纠纷6315件。落实人民调解三项经费，开展社会矫正、安置帮教试点工作，创建规范化司法所61个、民主法治示范村148个，"五五"普法通过省上验收。扎实开展社会治安综合整治"百日行动"，组建公安消防特勤队和应急救援支队，实施"天眼工程"和公安"三基"工程，全市治安形势保持平稳。强化食品、药品质量监管，重点对使用非食用物质、滥用食品添加剂和无证生产加工食品等不法行为进行整治，保障了群众健康安全。组织开展第六次人口普查，统计监测服务水平进一步提高。民族团结和双拥、边防工作深入推进，我市荣获全国人防先进城市称号。市防震减灾中心建成使用，妇女、儿童、台侨、宗教、档案等工作取得新的成绩。

　　8、落实惠民政策，社保体系不断完善。千方百计扩大就业，落实就业补贴资金9978万元，购买公益性岗位1222个，城镇新增就业1.4万人，城镇登记失业率控制在3.13%。组织开展"212"创业促就业行动，推行"创业培训＋小额贷款"新模式，新增小额贷款担保基金4200万元，为1.2万名创业人员和35户企业发放贷款5.5亿元，带动城乡就业5.7万人。稳步推行基本医疗、失业保险、工伤保险市级统筹，提高城镇职工、城乡居民基本医疗保险统筹基金支付限额和大病补助限额。推行新农合门诊统筹制度，住院报销比例提高5个百分点，参保人数57.8万人，参合率达到96%。调整企业退休人员养老金标准，月人均发放1256元，增加178元。城乡低保提标扩面工作完成，城市低保标准提高10%，农村低保年标准提高到900元，为城乡16.7万人发放保障金2.04亿元。在金塔、阿克塞推行新型农村社会养老保险试点，参保率均达到96%。村干部养老保险实现全覆盖，参保村干部1475名。启动实施被征地农民养老保险制度，参保农民1437人。五保供养标准达到每人每年2000元，保障人数3178人，集中供养率达到18.1%。实施城乡居民临时救助和医疗救助制度，为1.2万名临时困难人员发放救助金594万元，为6.8万人发放医疗救助金3221万元，"三无"老人、"五保"老人和低保老人全部纳入城乡医疗救助范围。健全完善"三老一少"补贴制度，为3.7万人发放特殊生活补助387万元。建立贫困寄宿生交通费、伙食费"双补"制度，1.3万名学生从中受益。继续实施农村饮水安全工程，又解决了5.74万农村居民的安全饮水问题。投资1.26亿元，新建、改建农村公路702公里。新建廉租房1250套，发放住房租赁补贴1.14万户，人均住房10平方米以下的低收入家庭全部纳入住房保障范围。实施农村危旧住房改造工程，改造危旧住房2.3万户、农村贫困残疾人危房450户。

　　9、靠实帮扶责任，移民开发成效显著。认真实施移民乡基础设施和经济社会发展两个五年规划，按照"规划先行、项目支撑、找准路子、强化措施"的要求，加快脱贫致富步伐，完善领导包挂、部门帮扶责任制，争取扶贫资金3.37亿元，移民贫困人口由6.45万人减少到5.82万人，下降9.8%。在瓜州县沙河回族乡召开项目帮扶现场会，落实帮扶物资和资金2325万元。因地制宜发展种植业，改良盐碱地2.3万亩，新建日光温室224座，经济作物面积达到14.2万亩，占农作物种植面积的61%。积极发展养殖业，调引良种羊2.2万只，新建暖棚圈舍2545座，牛、羊饲养量分别增长37%和11%。组织开展移民培训3.2万人，输转劳务2.1万人次，实现劳务收入8500万元。改善基础设施条件，营造农田防风林带1.96万亩，新建道路143公里，改建危旧住房1.2万户。启动农村低保和扶贫开发衔接试点，11个整建制移民乡低保补差标准和临时救助标准大幅度提高，发放移民低保、医疗救助、临时救助资金7000万元，受惠移民8.2万人。

　　10、加强自身建设，行政效能进一步提高。深入开展创先争优活动和"弘扬铁人精神年"活动，机关工作效率和服务水平进一步提高。完善重大事项集体决策制度，制定行政听证程序规定，组织召开城市供热、天然气入户等重大事项听证会，引导群众参与行政决策。修订完善市政府工作规则，制定市政府应急管理、政务督查和党组工作制度。规范政府采购、工程承包、土地出让等招投标程序，实现国土资源竞价收益5.7亿元。推行依法行政责任制，与33个政府部门、10个驻酒单位签订依法行政责任书，加大依法行政考核力度。深入推进政务公开和公用事业单位办事公开，行政服务中心受理办件36.5万件，办结率达到96.9%。新一轮机构改革全面完成，事业单位人事制度改革进一步深化。推进公务卡结算试点改革，完善国库集中支付制度，财政集中核算实现全覆盖。自觉接受人大法律监督、工作监督和政协民主监督，办理人大代表建议158件、政协委员提案161件，办结答复率达到100%。加强行政监察，强化对权、钱、人等关键环节和重点部位的监管，组织开展"小金库"专项治理，审计监督卓有成效。改善基层组织办公条件和村组干部待遇，新建、改建村委会办公用房50个，全市村、组干部年均报酬分别达到6000元和1800元以上，村办公经费达到5000元以上，均高于全省标准。

　　各位代表，2010年，我们克服了种种困难和严重的自然灾害，保持了全市经济社会平稳较快发展。这些成绩的取得，是我们贯彻落实中央和省委、省政府及市委战略部署的结果，是市人大、市政协和社会各界大力支持的结果，是全市人民辛勤努力、团结奋斗的结果。在此，我代表市政府向各位人大代表、政协委员和全市广大干部群众，向各民主党派、工商联、人民团体、离退休老同志，向驻酒人民解放军、武警官兵和公安干警，向中央、省属驻酒各单位以及所有关心、支持酒泉发展的各界人士，表示衷心的感谢和崇高的敬意！

　　在总结成绩的同时，我们也清醒地看到，酒泉的发展仍面临许多困难和问题，主要表现在：发展方式较为粗放，自主创新能力不强，资源环境代价较大；部分行业仍未摆脱困境，特别是矿产品采选冶炼效益下滑局面尚未扭转；地方税源不足，税收结构单一，财政收支矛盾加剧；农民持续增收难度加大，移民扶贫攻坚任务艰巨；蔬菜等基本生活必需品价格涨幅较大，城乡居民特别是低收入家庭生活负担加重；安全生产形势严峻，安全事故呈上升趋势；个别部门办事拖拉，服务意识、责任意识、创新意识不强，还不能完全适应科学发展的要求。这些问题，我们必须高度重视，在今后的工作中努力加以解决。

　　"十一五"主要成就及"十二五"任务目标

　　"十一五"时期，是酒泉发展史上极不平凡的五年。面对复杂多变的国内外形势，我们紧紧围绕加快发展这一主题，团结带领广大干部群众奋力拼搏，创造了"酒泉速度"和"酒泉奇迹"，提前一年完成了"十一五"规划目标。过去的五年是酒泉经济增长最快、发展质量最好、产业迅速壮大、人民普惠最多的五年。

　　--五年来，我们不断深化市情认识，将中央和省委、省政府的决策付诸于酒泉的实践，确立了"两抓整推"的总体思路，重点工作取得重大突破，难点工作取得新的进展，各项事业整体推进，实现了前所未有的跨越。风电装机达到550万千瓦，装备制造业销售收入达到223亿元。近两年累计争取省级移民扶贫资金4.67亿元，8.48万绝对贫困移民纳入低保范围，移民人均纯收入增速高于全市农民人均纯收入增速。全市经济总量突破400亿元，是"十五"末的2.8倍，以占全省3.9%的人口创造了全省9.9%的经济总量。财政收入突破50亿元，年均增长31.5%，高于经济增速8.9个百分点。

　　--五年来，我们加快优势产业开发，将培育区域经济竞争力作为强市富民的有力支撑，在工业发展上，坚持以风电开发为牵引，全面实施振兴工业"6+2"行动计划，地方工业实力明显增强，工业占生产总值的比重达到42.7%，比"十五"末提高8个百分点，对经济增长的贡献率达到54%；在农业发展上，大力推进"一特四化"进程，构建酒泉现代农业新体系，全市高效特色产业面积达到162万亩，占农作物播种面积的72.6%，肉羊饲养量达到565万只，饲养总量和农民人均饲养量均居全省第一；在三产发展上，坚持以线串点、以点带面，加快建设区域性商贸旅游中心，第三产业增加值达到140亿元，年均增长13.3%。累计接待海内外游客1552万人次、实现旅游收入109亿元，分别是"十五"期间的2.8倍和3.8倍。

　　--五年来，我们大力改善发展环境，将基础设施和生态文明建设作为酒泉可持续发展的关键，一大批铁路、公路、电力、水利、通讯、生态等重点项目相继建成，累计完成固定资产投资1104亿元，是"十五"时期的4倍。敦煌铁路、嘉安高速公路建成通车，完成农村公路建设6465公里，比"十五"期间增长2.4倍，100%的乡镇和98%的行政村开通班车。城乡面貌大为改观，城市建设累计投资达86.5亿元，城市建成区面积由73.2平方公里扩大到89平方公里，城市人均住房面积由20.5平方米增加到24.2平方米。农村砖木、砖混结构住房比重由33%提高到41.8%，城镇化率由55%提高到58.7%，全市城乡一体化水平总指数达到7.8，居全省第二。生态建设成效显著，关井412眼、压田4.5万亩，新增节水能力5000万立方米，森林覆盖率由1.87%提高到3%，增加1.13个百分点。全面完成节能减排约束性指标，万元GDP能耗累计下降20%。

　　--五年来，我们着力解决民生问题，把保障和改善民生作为政府工作的出发点和落脚点，政府累计购买公益性岗位6926个，发放创业促就业小额担保贷款6.6亿元，城镇新增就业5.5万人，下岗失业人员再就业2.4万人。社会事业全面发展，全市用于科教文卫体等方面的支出累计达50亿元，公共服务更加均等。城乡居民收入持续增长，分别比"十五"末增加5852元和2715元，年均增长10%以上，高于全国、全省平均水平。社会保障安全网更加完善，全市参加城乡养老、医疗、失业、工伤等各类保险的参保人数达到114万人次，社会保险覆盖面达到85.3%；为城乡低保户、"三老一少"、离任村（社区）干部、低保边缘户、五保户发放救助资金6.9亿元，城乡困难群众得到了更多的实惠。累计完成投资1.56亿元，解决了27.8万人的安全饮水问题。市政府五年来承诺的48件惠民实事得到较好落实。

　　"十二五"时期，是全面建设小康社会的关键时期，是实现酒泉经济跨越式发展、加快推进强市富民进程的重要时期。国家大力支持低碳经济发展，并将我市定位为新能源开发利用示范区、风电和太阳能发电示范基地、风电装备生产基地、光伏和光热产品研发制造基地，这为我市新能源及装备制造业发展描绘了广阔的前景。国家新一轮西部大开发战略及支持甘肃发展的47条意见，为我市实现跨越式发展提供了良好的政策机遇。国家扶持新疆发展的优惠政策，将进一步凸显我市的地域优势，为我市发展通道经济创造了有利条件。省委、省政府把我市确定为"两翼齐飞"的重要一翼给予扶持，更加坚定了全市广大干部群众率先发展、加快发展的信心。我们正在打造的"6+2"现代工业体系、"一特四化"现代农业体系和区域性商贸旅游中心，将为我市"十二五"发展奠定坚实的基础。这些有利条件表明，我们完全可以发展得更快、发展得更好，率先实现全面建设小康社会的宏伟目标。

　　"十二五"时期的总体要求是：以科学发展观为指导，全面落实党的十七大和十七届五中全会精神，认真贯彻省委、省政府"中心带动、两翼齐飞、组团发展、整体推进"的区域发展战略，按照市委"两抓整推"总体思路，以提高人民群众生活水平为目标，以加快转变经济发展方式为主线，以改革开放和科技创新为动力，以重大项目为支撑，大力实施振兴工业"6+2"行动计划，加快推进"一特四化"现代农业，发展文化旅游产业和通道经济，强化基础设施和生态环境建设，提高社会公共服务水平，保障和改善民生，努力实现全市经济社会跨越式发展。

　　"十二五"时期的奋斗目标是：到2015年，全市生产总值、工业增加值、财政收入、固定资产投资、社会消费品零售总额五项指标比"十一五"末翻一番；城镇居民人均可支配收入年均增长13%，农民人均纯收入突破1万元，年均增长11%；万元生产总值能耗下降20%，经过五年的努力，把酒泉建成全国重要的新能源及新能源装备制造基地、资源综合利用基地和低碳经济综合示范区。

　　为实现上述目标，我们要准确把握酒泉的市情和阶段性特征，找准我市在全省区域发展战略中的定位，坚持不懈地抓好重大项目建设，打造和提升"6＋2＋3"产业体系，切实将科学发展的理念转化为干部群众的自觉行动，将中央和省委、省政府的决策付诸于酒泉的具体实践。

　　--坚持项目带动。抢抓国家政策机遇，充分发挥项目建设在扩总量、转方式、调结构、惠民生等方面的支撑作用，着力抓好基础设施、生态保护、产业开发、社会发展等方面的重大项目，努力将我市建成政策倾斜的"洼地"和产业西移的最佳承接地，按照引大做强的要求，创新招商引资方式，提高招商引资质量，力争固定资产投资年均增长30%以上。

　　--强化产业支撑。以"6+2+3"产业建设为重点，加快新型工业体系建设、"一特四化"现代农业建设和现代服务业发展，高度重视通道经济在产业融合、扩大协作等方面的重要作用。发挥新能源建设的"龙头"作用和中小企业的基础作用，力争"十二五"末全市风电装机达到2000万千瓦，光电装机达到2000兆瓦，新能源装备制造业销售收入突破500亿元，工业增加值年均增长20%以上。夯实农业基础地位，提高农业产业化经营水平，努力实现农业增效、农民增收和农村环境面貌显著改善的目标，高效节水面积达到210万亩，新增108万亩，农民人均纯收入保持两位数增长。抓好国家服务业综合改革试点工作，提高服务业的比重和质量，将文化旅游业培育成带动经济发展的战略性新兴产业，扩大消费需求，促进经济增长由投资拉动转向投资、消费双轮驱动。

　　--优化发展环境。加强基础设施和生态环境建设，提高资源保障水平和可持续发展能力。加快推进城镇化进程，构建以酒泉市区为中心、6个县市为纽带、11个建制镇为节点的城镇体系，新增城市人口5万人，城镇化率提高5个百分点。改善交通基础设施，构建"四横四纵"铁路体系和"2229"机场体系，力争100%的乡镇通四级以上公路，95%的行政村通油路。构筑生态安全屏障，强化价格引导机制，落实农业、工业、工程、生活、中水回用五大节水措施，以实施敦煌水资源综合利用和生态保护规划为契机，抓好西片五县市节水工程建设，积极争取扶持政策，推进肃州、金塔节水工作，建设国家级湿地补偿试点、生态补偿实验区、荒漠化治理示范区，森林覆盖率提高2个百分点，达到5%。按照"淘汰落后、严控两高、加强管理、强化监督"的要求，抓好工业、建筑、交通三大重点领域节能和重点行业减排，确保完成"十二五"节能减排任务。

　　--着力改善民生。更加注重社会建设，切实保障和改善民生，按照"学有所教、劳有所得、病有所医、老有所养、住有所居"的要求，深化公共服务领域改革，构建覆盖城乡的公共服务体系，推进公共服务均等化，使发展成果惠及全体人民。完善就业、养老、医疗、低保、救助等制度，基本建立起广覆盖、保基本、多层次、可持续的社会保障体系，城镇登记失业率控制在4%以内。提高人民生活质量和水平，增加低收入家庭收入，扩大中等收入群体比重，坚持不懈地抓好移民扶贫开发，力争"十二五"末移民人均纯收入达到全省农民人均收入水平，缩小市民、农民、移民收入差距。

　　2011年政府工作

　　今年是实施"十二五"规划的第一年，做好今年的工作，对于"十二五"开好局、起好步，奠定跨越式发展的基础，具有十分重要的意义。

　　全市经济社会发展的预期目标是：地区生产总值增长16%，固定资产投资增长30%，财政收入增长16%，社会消费品零售总额增长20%，城镇居民人均可支配收入增长13%，当地农民人均纯收入增长11%，移民人均纯收入增长20%，城镇登记失业率控制在4%以内，万元生产总值能耗降低4%，主要污染物排放完成省上下达的约束性指标，人口自然增长率控制在5.5‰以内。实现上述目标，应重点抓好八个方面的工作：

　　1、加快构建"6＋2"工业体系。以风电开发为牵引，加快推进振兴工业"6＋2"行动计划，着力抓好风电二期800万千瓦和65万千瓦大型国产化风机示范项目建设，新增风电装机300万千瓦。争取200兆瓦光电和金塔50兆瓦光热发电项目开工建设，力争全市光电装机达到200兆瓦。继续争取风电、光电税收优惠政策，培植地方财源，加大新能源基地配套设施建设投入。加快明沙窝、常乐、红柳洼、国电酒泉热电厂二期和昌马电厂前期工作。新建、续建水电站5座，新增水电装机6万千瓦。破解电力输出瓶颈难题，做好±800千伏特高压直流输变电工程前期工作，加快马鬃山110千伏和柳园、敦煌330千伏变电站建设。提高装备制造业水平，实现大容量主流机型批量生产，抓好关键零部件配套生产；引进光电装备制造企业，形成100兆瓦光伏组件能力，风光电装备制造业销售收入突破300亿元。依托新能源基地建设，加快发展高载能产业，推进煤炭、黄金、石棉等矿产资源整合，促进资源节约、集约利用。加强与东中部地区的合作，探索建立"飞地"园区，加快承接产业转移。发挥民营经济在扩大就业、繁荣市场、培植税源等方面的重要作用，认真落实国扶民营经济36条政策，扶持民营经济做大、做强、做活，新培育销售收入亿元以上工业企业5户、千万元以上工业企业20户，力争工业增加值增长20%以上。

　　2、推进"一特四化"现代农业进程。以农业转型升级和促进农民增收为重点，大力发展"一特四化"现代农业，加大强农惠农力度，完善以奖代补措施，财政支农资金增长10%以上。调整优化种植结构，建成市级农业科技示范园区20个，新增3000元以上高效田13万亩。扶持沿山片区发展特色产业，拓宽农民增收渠道。继续推进牛羊产业大县建设，扶持奶牛产业发展，调引良种羊2.2万只，新增肉羊50万只。做大做强农产品加工龙头企业，培育农民专业合作示范社10个，农产品贮藏加工增值率达到50%以上。开展农业科技培训20万人次，完成农民技能培训2万人，输转农村劳动力15万人次。加强耕地涵养保护，完善农产品质量安全监管监测体系，推行农产品市场准入制度，农产品质量安全合格率达到98%以上。健全县乡村土地流转服务体系，规范农村土地经营权流转。大力推进新农村建设，继续抓好"一池三改"、村镇基础设施建设、村组道路硬化和农村危旧房改造，整治提升村容村貌示范点100个，新建社区化中心村20个。深入实施移民开发两个五年规划，争取落实各类扶贫资金1.5亿元，改良土地3万亩，发展特色种植2万亩，实现移民减贫6000人。

　　3、抓好重大项目建设。加大项目规划、策划投入，加快重大项目建设，推进瓜星高速、敦当公路、兰新铁路第二双线建设进度，开工建设酒航铁路、敦格铁路、"三北"通道酒泉段和桥红公路，抓好酒航高速、瓜敦高速项目前期工作，完成敦煌机场扩建工程和酒嘉城际一级公路路基建设，建成敦煌公路客运枢纽站。加强生态环境建设，以防风沙、培水源、造景观为重点，全面启动北大河生态综合治理项目，继续实施中小河流治理、水库除险加固等水利工程，完成洪临、党河、白石灌区续建配套年度任务，争取启动敦煌水资源合理利用与生态保护项目，"引哈济党"工程开工建设。加强交流合作，扩大对外开放，促进外经贸稳定增长，积极筹备参加"兰洽会"等重大招商节会，力争引进到位资金200亿元以上。

　　4、增强城镇聚集辐射功能。以建设"殷实、敦厚、低碳、宜居"的戈壁绿洲城市为目标，按照"中建西拓"的思路，加快新城区与工业园区连接带建设，向西拓建环保、低碳、零排放的工业园区，优化酒嘉一体化空间布局。编制城市用地储备规划，修编上报酒泉城区总体规划，实现全市乡镇总体规划全覆盖，村庄规划覆盖率达到95%以上。着力提升城市综合承载能力，加快已拆迁片区的开发建设进度，完善道路、供水、供气、供热和垃圾处理等市政设施，抓好污水处理、雨水分离收集和中水回收利用，提高城市水资源利用率。实施中心城区增绿工程，新增城市公共绿地54.6万平方米。推行城市扁平化、精细化、规范化管理，加快"数字城管"建设，实现城市管理与城市建设同步跟进、无缝覆盖。深入开展市容环境综合整治，积极创建全国文明城市。研究制定有利于城市化发展的就业、居住、社保、户籍政策，促进农村人口向城市转移，新增城市人口1万人，城市化率提高1个百分点。

　　5、促进现代服务业和通道经济发展。全面启动实施国家服务业综合改革试点工作，形成生产与服务并重、投资与消费互动、现代服务业与传统服务业融合的发展格局，力争第三产业增加值增长14%以上。以过境干线、管道和电力主网建设为依托，大力发展通道经济和现代物流业，加快酒嘉物流园区建设，抓好新能源配套服务，组建新能源研发中心和风光电设备检测认证中心。提高金融服务质量，争取交通银行、招商银行在我市设立分支机构，启动筹建酒泉银行的前期工作。扶持发展科技、信息、咨询、担保、法律等中介服务组织，大力提升餐饮娱乐、商贸流通、维修等传统服务业水平。继续实施"万村千乡"和"双百市场"工程，推进社区、乡镇商业网点建设，做好家电、摩托车下乡工作，落实家电以旧换新政策，保持消费持续增长。加快旅游业发展，打造"中国飞天之都"新形象，努力构建三大品牌引领、两大旅游圈支撑、六条精品线路串联的"326"旅游发展体系。扩充旅游发展基金，扶持重点旅游项目，开工建设酒泉酒文化博览园、敦煌阳关景区二期工程，加快开发风电旅游项目，推进敦煌旅游上市，争取将鸣沙山·月牙泉创建为5A级景区，拓展周边及国内外旅游市场，力争接待海内外游客500万人次以上，增长23.5%，实现旅游综合收入46亿元，增长22%。

　　6、加强生态环境保护。严格执行"三禁"政策，加大资金扶持力度，实行差别水价制度，加紧实施百万亩节水灌溉工程，提高节水示范园区建设水平，新增节水高效灌溉面积2万亩。继续实施封滩育林、禁牧休牧工程，抓好祁连山水源涵养区保护及疏勒河、党河、黑河流域治理。广泛开展全民植树活动，完成人工造林11万亩，义务植树320万株，全面完成集体林权改革任务。加快移民区农田林网建设和村镇绿化进度，促进移民区生态修复。落实节能减排目标责任，推广资源节约、替代和循环利用技术，淘汰落后产能。抓好工业园区、开发区及企业达标治理，确保完成节能减排任务目标。落实农村"以奖促治"政策，扎实开展环境优美村镇创建活动，抓好22个规模化畜禽养殖场的环境综合整治，加强农业环境保护，废旧农膜回收加工利用率达到60%以上，积极创建国家环保模范城市。

　　7、统筹发展社会事业。强化社会事业的公益性、普惠性、均等性，满足广大群众对公共服务的需求。加大教育投入，力争教育支出占财政支出比重达到15%以上，提前一年达到省上要求。做好新增教育资源布局规划，加大城区建校力度，缓解过大班额，实施校舍安全工程，制定学前教育扶持政策，改善移民乡、农垦团场办学条件，开工建设敦煌四中。加强学校精细化管理和校园安全管理，建成市级标准化学校20所。拓展酒泉新能源技术学院合作办学领域，建立健全新能源技术创新体系、检测认证体系和科技服务平台，提高新能源及装备制造业产、学、研水平，增强自主创新能力和示范带动效应，继续实施科技特派员基层创业工程，加快科技创新和成果转化。按照保基本、强基层、建机制的要求，深化医药卫生体制改革，完善国家基本药物制度。完成市医院门诊大楼及6家县级医院标准化建设任务，提高市医院床位补贴标准。完善突发公共卫生事件应急处置措施，高度重视甲型流感、鼠疫等重大疾病防控，积极创建全国卫生城市。深化人口与计划生育综合改革，促进人口均衡发展，开展"生殖健康服务进万家"行动，加强移民和流动人口计生管理。开工建设"六馆合一"的酒泉文化中心，加快基层文化站室建设，新建文化信息资源共享工程村级服务点20个，"农家书屋"实现村级全覆盖，审定出版《酒泉通史》。举办第三届青少年田径赛、第三届敦煌沙滩排球赛和玄奘之路戈壁挑战赛。全面完成"村村通"建设任务和玉门、瓜州数字电视整体转换工程，广播电视覆盖率达到100%。制定少数民族和民族地区跨越发展实施意见，继续开展民族团结进步宣传月活动，促进民族团结与宗教和顺。开展双拥创建和军民共建活动，推进残疾人社保和服务体系建设，建成瓜州、阿克塞残疾人抚养机构，做好统计、档案、外事、人防、边防及防震减灾工作。

　　8、着力保障和改善民生。按照"普惠、实惠、常惠"相结合的原则，提高民生保障水平。实施更加积极的就业政策，通过项目带动就业、创业促进就业、政府援助就业、培训提升就业等途径，努力扩大就业规模。切实做好大学生就业工作，采取政府扶持与企业引导相结合，鼓励大学生到企业就业，促进劳动力就业本地化。对零就业家庭、残疾人员、低保对象和下岗失业人员实施重点援助，提高环卫工人工资标准，购买公益性就业岗位1000个，新增城镇就业1万人以上。健全完善覆盖城乡的社会保障体系，全面落实城镇职工基本医疗、工伤保险、失业保险市级统筹，探索建立城镇居民基本医疗门诊统筹制度，巩固城乡低保提标扩面成果。积极探索城乡养老社会化新机制，推进城市养老院、农村敬老院建设，继续完善村干部养老保险和被征地农民养老保险制度。坚持"阳光救助"，提高部分优抚对象补助标准，完善"三老一少"生活补贴制度、家庭困难寄宿生"双补"制度、被辞退民办教师生活补助和临时救助制度，落实城乡医疗救助实施办法。开工建设人力资源市场和社会保障服务中心，加快建设城市综合福利服务中心、救助管理站和流浪儿童救助保护中心。把平抑物价作为宏观调控的重点，积极探索地价房价综合招标制，加强房地产市场监管，扩大保障性住房供给，抑制房价过快上涨。设立"菜篮子"基地建设扶持基金500万元、农产品市场价格调节基金100万元，扶持发展日光温室4000亩，新增设施蔬菜1万亩，在社区建立惠民肉菜店，鼓励农超对接、产地直销，保证广大群众吃上廉价、放心的蔬菜。严格落实安全生产责任，开展重点领域专项整治，严防重特大事故发生。加强食品、药品、饮食卫生安全监管，确保人民群众身体健康。深入推进社会矛盾化解、社会管理创新、公正廉洁执法，建立重大社会决策、重大工程项目社会稳定风险评估机制和多元化矛盾纠纷解决机制，畅通群众利益诉求渠道，从源头上预防和减少信访问题的发生。启动"六五"普法工作，完善"三调联动"工作体系，积极开展法律援助，保障弱势群体合法权益。深入实施公安"五大建设"和"三基"工程，加强城乡社区警务建设，完善社会治安防控体系。加大社会管理薄弱环节整治力度，严厉打击各类违法犯罪活动，保障人民生命财产安全和社会大局稳定。

　　切实解决民生问题，办好十件惠民实事：一是实施"223"创业促就业行动计划，新增小额贷款担保基金2000万元，发放贷款2亿元，带动就业3万人以上；二是实施校舍安全工程，改造危旧校舍3万平方米；三是搭建新农合信息平台，实现新农合门诊刷卡报销，解决好36家社区卫生服务中心（站）人员工资和运行经费问题；四是继续实施农村安全饮水工程，解决5万名农村居民安全饮水问题；五是新建、改造农村道路1000公里；六是修建保障性住房3000套（户），其中廉租房1000套、公共租赁房1000套、棚户区改造1000户，发放租赁补贴1万户；七是改造农村贫困群众危房2.2万户、残疾人危房450户，改建50个村级组织危旧办公用房；八是巩固金塔、阿克塞新型农村社会养老保险试点成果，启动肃北试点工作，60岁以上农村老人人均发放月基础养老金55元，参保率达到95%以上；九是政府租赁门点，在10个社区设立惠民肉菜店，惠民肉菜店覆盖率达到50%以上；十是加快酒泉热电联供和天然气入户进度，年内实现城区供热380万平方米，3万户居民实现通气。

　　各位代表，做好"十二五"开局之年的各项工作，意义十分重大。我们一定要扎实推进政府自身建设，改进工作作风，提高科学决策水平，完善重大事项专家咨询、公开听证、法律论证和集体决策制度。加强行政执法监督，强化职权行使监督和问责追究。继续推进政务公开，快捷办理审批事项，强化政府服务功能。认真履行党风廉政建设职责，注重从源头上预防和治理腐败，严格执行土地出让、工程承包、政府采购、产权交易等招投标程序，深化经济责任、政府投资项目、民生资金、财政、资源和环境审计，确保权力在阳光下运行。

　　各位代表，"十二五"是实现酒泉经济跨越式发展的关键时期，面对新的目标、新的使命和广大群众新的期待，我们深感任务艰巨、责任重大。让我们在中共酒泉市委的坚强领导下，团结带领全市广大干部群众，攻坚克难，奋力拼搏，为全面完成市二届人大七次会议确定的任务目标而努力奋斗！
